# The Health Effects of Karate Training: A Review of 21st Century Research

**DOI:** 10.3390/healthcare13020118

**Published:** 2025-01-09

**Authors:** Paweł Adam Piepiora

**Affiliations:** Faculty of Physical Education and Sports, Wroclaw University of Health and Sport Sciences, 51-612 Wrocław, Poland; pawel.piepiora@awf.wroc.pl

**Keywords:** bunkai, health, karate, kata, kihon, kumite

## Abstract

**Background/Objectives:** To date, the health effects of karate have not been identified. Therefore, the aim of this article is to learn more about the health effects of karate training based on a review of current research. **Methods:** The Scopus database was searched from 2000 onwards for available articles related only to karate. The following intermediate phrases were not included: combat sport, fighting art, hand-to-hand combat, martial art, and self-defence system. The filter used was desk review analysis. Only 14 scientific articles (research papers and review papers) strictly on karate were found. The method of source material analysis and critical analysis of the source text was then used. **Results:** It was shown that kumite appears to require significantly more metabolic power than kata. Furthermore, the greater skill of karatekas is associated with their greater physical fitness, and long-term karate training attenuates the decline in dynamic visual acuity (DVA). The role of modified and individualised forms of karate training is also highlighted as important for the health of practitioners. Furthermore, long-term karate practice is associated with extensive modulation of immunological parameters. Karate training can also significantly improve motor skills. It can also play an important role in the development of willpower and personality traits that contribute to the well-being of its practitioners. Furthermore, nutritional and psychological interventions combined with karate training may improve cardiometabolic parameters, oxidative stress and inflammation. Karate training may also contribute to the prevention of osteoporosis and strengthen the left ventricular myocardium. **Conclusions:** It was found that there is a paucity of contemporary research on the health effects of karate training. In addition, they are limited to the individuals studied, so that generalisations about these effects in the general population of karate practitioners cannot be made.

## 1. Introduction

Early masters predicted that the usefulness of this fighting method would continue to decline as karate grew in popularity [1]. However, thanks to its robust masters, karate actually developed worldwide in the 20th century [2]. Today, the undeniable utility of karate is that it is a combat sport, martial art, and self-defence system all in one [3]. However, the contemporary decline in popularity of karate, like that of other martial arts, is linked to the escalation of neo-gladiatorialism, better known as mixed martial arts [4]. In contemporary societies, ‘martial arts’ has become synonymous with ‘mixed martial arts’ [5]. This is a problem because the term ‘martial arts’ is used to refer to the training of a particular fighting method to maintain health, whereas ‘mixed martial arts’ have nothing to do with health [6]. For this reason, martial arts that are anti-violent in premise are moving away from the term ‘martial arts’ to seek another precise identification [7,8].

The advantages of karate training are manifold. They include the potential for practitioners to experience eastern relaxation techniques and the opportunity for active rest and relaxation [9]. Furthermore, the systematic training of the body and mind provides opportunities for self-realisation and improvements in physical and mental health [10]. Additionally, it is possible to identify individual short-term objectives, the accomplishment of which is contingent upon the individual in question [11]. In addition, the importance of learning to maintain attention, inner calm, and emotional control is underscored [12]. Additionally, practical knowledge of self-defence techniques and how to help oneself and others is also emphasised [13]. Moreover, the acquisition of knowledge regarding a different culture and philosophical tradition is of significant value, as it fosters the development of tolerance and respect for diverse perspectives and individuals [14]. It is also important to function in a group with a defined hierarchy, to monitor one’s own and others’ progress, and to recognise one’s shortcomings [15]. Additionally, opportunities exist for participation in sporting competitions on a basis of fair play, with the objective of testing one’s abilities [16]. Habits are developed to overcome difficulties through perseverance and systematic work on oneself [17]. One learns how to learn from failures by turning them into future successes [18]. This implies satisfaction in achieving successive training degrees, which in turn gives moments of joy from sports trophies. Furthermore, it acts as a mobilising factor to achieve success in other areas of life [19].

It is important to note, however, that the aforementioned health benefits of karate training are not unique to this practice but are also observed in the training of other fighting methods. The aim of this article is, therefore, to provide a detailed account of the pro-health effects of karate training relating to the benefits of maintaining physical and mental health. To this end, research works on karate from the 21st century have been reviewed. A research search revealed that there has been no comprehensive coverage of the health effects of karate training in the past twenty-four years. Accordingly, a niche has been found that is relevant to physical culture sciences in the area of combat sports. This is the first work of its kind where the pro-health effects of karate training have been distinguished from the generalisations of combat sport, fighting art, hand-to-hand combat, martial art, and self-defence system.

## 2. Material and Methods

### 2.1. Data Sources

A search of the Scopus database was conducted for articles published from the year 2000 onwards that were strictly related to karate. This database was chosen because it has the largest global coverage in relation to the databases PubMed and Web of Science. In this sense, Scopus is the world’s largest database indexing exclusively peer-reviewed scientific publications, conference reports, and books, as well as patents. In total, more than 38,000 journals have been indexed in the database, while about 23,000 are currently active. The difference is due to the fact that some periodicals have ceased operations, merged with others, changed their title, or been removed by the board that oversees the quality of indexed journals [20]. This does not mean that the searched articles are indexed together in the databases PubMed, Scopus, and Web of Science.

### 2.2. Search Strategy

The filter used was desk review, which consisted of verifying keywords in documented data in the Scopus database [21]. Desk review is used to provide an overview of the current state of knowledge in an area [22]. The following keywords were used in the searches: bunkai, effects, health, karate, kata, kihon, kumite, pro-health, and training. At the same time, only articles in English were included, due to their international reach [23]. Articles that made indirect references to karate by categorically identifying the subjects as practitioners were excluded from the search results. This included, but was not limited to, the following terms: combat sport, fighting art, hand-to-hand combat, martial art, and self-defence system. This was important because many researchers include subjects from different combat methods in their work. And thus inferences about karate alone are not possible. The search was conducted on 9 September 2024.

### 2.3. Inclusion and Exclusion Criteria

Only studies that relate strictly to karate were included, regardless of the used methodology and article type (research article, original article, review article, perspective, mini review, hypothesis and theory article, and other types). Articles not strictly related to karate were excluded, for example those that captured subjects from karate and other fighting methods in a single sample.

### 2.4. Article Selection and Quality Assessment

A search of the literature revealed that only 14 articles dealing explicitly with karate were published in the 21st century. The retrieved articles meet the timeliness criteria and rigorous source selection criteria of the Scopus database. Prior to publication in the Scopus database, an independent panel of experts (Scopus Content Selection and Advisory Board) evaluates journals and books to be included in the Scopus database. Minimum criteria include the following: international reach of the journal, clearly defined objectives and scope of the subject matter, consistency of editorial board composition with the subject matter, regularity of publication, assigned ISSN, abstracts, titles and keywords in English, good quality, complete website in English, references in Latin, access to online content, and having and adhering to a Publishing Ethics and Malpractice Statement.

### 2.5. Bias Assessment

To assess the risk of bias in the selected studies, a method of source material analysis and critical analysis of the source text was employed. The methods of analysing source materials and critical analysis of the source text are used to demonstrate the originality of the problem situation chosen for study and the desirability of analysing it [24]. These methods refer to the verification of what already exists in the literature and what is known, and to prove the necessity of investigating unknown hypotheses [25]. The effect of using these methods is to show the relationships, differences, and dependencies of the studied topic with the existing state of knowledge. The essence is to show the value of the subject in the existing knowledge [26].

This review did not require ethics committee approval.

### 2.6. Transparency and Reproducibility

The adopted procedure described in the subsections follows the methodology of qualitative research in article search, selection, and analysis.

## 3. Health Effects of Karate Training

In the first available study, Doria et al. [27] verified the rate of oxygen uptake with inhalation (VO_2_, L min^−1^) and the concentration of blood lactate before and during exercise and recovery among 6 kata and 6 kumite champions who competed in simulated competitions. The present study revealed no significant differences between the kata and kumite groups with regard to VO_2_max, vertical jump height, and Wingate test. However, the kumite masters group exhibited significantly higher metabolic power than the kata masters group. There were significant differences between genders and competitors in the percentage of lactic acid derived from oxygen and anaerobic sources, while the source of lactic acid itself was similar. Based on these findings, it can be concluded that kumite requires a significantly higher metabolic power than kata, with oxygen being the dominant energy source.

In another study, Chaabène et al. [28] conducted a review of the most salient physical and physiological characteristics of karate athletes. These were quasi-experiments on samples of up to 30 people. It was observed that male karate practitioners at the highest competitive level exhibit low levels of body fat and a mesomorphic/ectomorphic somatotype, with aerobic capacity being a significant factor. It can be stated that there is no significant difference between men and women training in kata and kumite with regard to aerobic capacity. However, there is a discrepancy in anaerobic capacity, specifically in the maximum power demonstrated by karatekas in the power-velocity test, between competitors at the national and international levels. Conversely, no longer is there a distinction between the two in the maximum cumulative oxygen deficit test. Moreover, the capacity for muscular explosive power is a significant factor in the ability of karate athletes to perform at a high level. Moreover, karate performance is more dependent on muscular strength at lower loads than at higher loads. However, there is a notable discrepancy in fitness levels between highly advanced and novice male karatekas. Additionally, the ranges of bilateral hip and knee flexion among karate athletes are greater than those observed in non-karate athletes. There is also a significant difference in reaction time between high-level karatekas and beginners. Based on these observations, it can be concluded that the superior skills of karate athletes are related to their greater physical fitness.

In a subsequent study, Muiños and Ballesteros [29] investigated the benefits of intensive, long-term karate training among 30 young and 15 older athletes in a quasi-experiment designed to verify the mitigation of age-related decline in dynamic visual acuity (DVA). It was observed that karate athletes exhibited superior DVA compared to non-athletes. The older adult groups exhibited a more pronounced oblique effect than the younger groups. Moreover, age-related changes in athletic performance were observed in the high-velocity condition. The DVA of young karate athletes was superior to that of non-athletes, and older karate athletes exhibited enhanced DVA compared to older individuals with a sedentary lifestyle. Based on these findings, it can be posited that long-term karate practice attenuates the decline in DVA, indicating neuroplasticity in the ageing human brain. Therefore, among the elderly, it is crucial to practice karate in general rather than precisely targeting kata or kumite.

Later, Mastnak [30] conducted a narrative review and a phenomenological synthesis. He concluded that karate training has a beneficial effect on the musculoskeletal system, reducing muscle tension and helping to adjust posture. It can therefore be surmised that karate training has a beneficial effect on the prevention of work-related overload syndromes and musculoskeletal diseases. This indicates that karate training can act as a risk-reducing factor for work-related overload. Furthermore, the role of modified and individually adapted forms of karate training, through the adaptation of training standards and the ability to self-regulate them, as relevant to health, is also highlighted.

In a subsequent study, Manzaneque et al. [31] investigated the impact of karate training on immunological parameters. The subjects were 15 karate athletes with a minimum of three years of training experience, undertaking training three days per week, and 12 volunteers, whose blood samples were obtained for the purpose of quantifying immunological parameters. The karate athletes exhibited significantly elevated levels of total leukocytes, monocytes, and lymphocytes, along with a heightened proportion of monocytes and elevated serum IgG and IgM concentrations. Based on these observations, it was postulated that prolonged karate training exerts a profound influence on the modulation of immunological parameters, encompassing total and specific leukocyte counts, as well as immunoglobulin concentrations.

In a separate study, Altavilla et al. [32] estimated the effects of four weeks of karate training among male adolescents aged 14 to 16 years. The results demonstrated a notable enhancement in the motor competencies of 12 karate athletes, as evidenced by medicine ball throw, shuttle run, and long jump tests, in comparison to 12 untrained male subjects. Therefore, the hypothesis that karate training can significantly influence motor abilities was supported.

In a subsequent study, Rutkowski et al. [33] evaluated alterations in individual physical fitness tests among 59 early school-aged children engaged in systematic karate training in a quasi-experiment. Eurofit tests were conducted before and after ten weeks of karate training. The results demonstrated positive changes in physical fitness among the children tested. Statistically significant improvements were observed among children with normal body weight compared to children who were overweight or obese. The findings suggest that karate training significantly shapes children’s physical fitness.

A subsequent study by Shaw et al. [34] validated the isokinetic strength and power of the quadriceps and popliteal tendon of the lower body, as well as the hip and knee flexibility of karate athletes (including 18 elite karate athletes and 18 advanced karate athletes; relative to non-trainees). The findings indicated that advanced karateka athletes exhibited a notable enhancement in hip flexion, hip extensors, and hip rotation. In contrast, elite karateka athletes exhibited the greatest strength in the quadriceps and popliteal tendons, and demonstrated significantly higher quadriceps peak torque values than non-trainees. Additionally, elite karate athletes exhibited a shorter time to peak torque for the quadriceps compared to advanced karate athletes. Based on these findings, it was concluded that karate training at a high level is associated with generating more force and a reduced flexibility range.

In another study, Naserpour et al. [35] verified knee injury profiles among 390 Iranian elite karate athletes using knee injury information obtained from the Knee Outcome Survey. It was observed that the majority of karatekas experienced knee injuries, including anterior cruciate ligament (ACL) rupture, articular cartilage damage, and meniscus damage. Furthermore, no differences in knee injuries were identified between dominant and non-dominant limbs. The majority of injuries occurred during the preparation periods for competitive events. The results indicate that karate training at a high competitive level is associated with a high probability of knee injuries and trauma, which translates into the negative health effects of karate training.

In a further study, Potoczny et al. [36] verified the existence of an indirect effect of self-control and emotion regulation on the relationship between karate training and life satisfaction in a quasi-experiment among 58 karate practitioners in relation to 59 non-training subjects. The results demonstrated that there was no direct effect of karate training on life satisfaction. However, there was an indirect relationship between karate training and life satisfaction through self-control and the re-evaluation of priorities. This suggests that self-control and the re-evaluation of priorities fully mediate the effects of karate training on the subjective well-being of practitioners. Consequently, the hypothesis that karate training can play an important role in shaping volitional and personality traits that contribute to participants’ well-being is supported.

In a separate study, de Souza et al. [37] investigated the impact of 12 weeks of karate training, three times per week, on cardiometabolic parameters, oxidative stress, and inflammation among 35 overweight and obese adolescents in relation to 35 non-trainers. The results demonstrated that the karate group exhibited a reduction in resting heart rate, high-density lipoprotein cholesterol, and triglycerides, and an increase in low-density lipoprotein cholesterol in comparison to the baseline measurements prior to the commencement of the experiment. Furthermore, a reduction in protein carbonylation and nitric oxide was observed, while superoxide dismutase, glutathione, and adiponectin exhibited an increase in this group in comparison to the pre-experimental condition. The findings suggest that the combination of nutritional and psychological interventions with karate training can enhance cardiometabolic parameters, oxidative stress, and inflammation in overweight and obese adolescents.

Later, Glinkowski et al. [38] validated heel bone assessments among 44 karate practitioners, including 24 black and 20 coloured belts, utilising a quantitative ultrasound method as an indicator for assessing bone fracture risk. Significantly higher bone density was found among black belt holders. Furthermore, long-term trainees achieved results that were significantly above the norm for their age groups. Based on these findings, it was concluded that long-term karate training can significantly affect the parameters of quantitative ultrasound measurements of the heel bone. Thus, it may be posited that karate training may be helpful in the prevention of osteoporosis. However, further clinical research is needed to substantiate this.

In a separate study, Gaweł et al. [39] evaluated the long-term effects of kata training on posture and musculoskeletal pain (range of motion) among 12 elite karate athletes. It was observed that age and the number of weekly kata sessions were correlated with lumbar spine range of motion (ROM). A notable increase in the prevalence of lumbar hypolordosis and posterior pelvic tilt was observed following karate training sessions. There was a significant difference in the range of motion of tilt in the sagittal plane between the first and second trials, with an average difference of 10.0 degrees. It can therefore be concluded that the kata postures and their movement pattern are related to the presence of abnormal hip internal and external rotation and reduced lumbar lordosis depth, pelvic tilt, and their range of motion. This leads to the hypothesis that the locations of long-term musculoskeletal complaints (NMQ-6) result from compensatory changes occurring in musculoskeletal structures as a result of kata training at the elite level.

In the most recent study, Skorosov et al. [40] investigated the impact of regular karate training on the cardiac characteristics of individuals who had been engaged in training for a period of one year and who had practised karate on three occasions per week for a minimum of six months. The researchers conducted ultrasound diagnostics of the cardiac condition and observed a tendency among 15 karate trainers to develop left ventricular hypertrophy of the myocardium, characterised by an increase in mass and an expansion of the walls in relation to 16 non-trainers. The external dimensions of the left ventricle and the volume of its cavity among karate athletes remained within the normal range, although there was a slightly higher rate of myocardial relaxation than in the comparison group. It was therefore concluded that karate training for a minimum of six months can strengthen the left ventricular myocardium, maintaining its optimal internal volume, external dimensions, and functionality.

A summary of the results from this review is provided in Table 1.

## 4. Discussion

### 4.1. Explanation of the Subject Matter

The positive effects of karate training on health have been documented, as in other fighting methods, in the results of anthropological [41], anthropomotor [42], biological [43], medical [44], and psychological studies [45].

Taken as a whole, the findings indicated that karate training was linked to enhancements in muscular fitness, lower body strength, power, and flexibility. It has been demonstrated to exert a beneficial influence on cardiometabolic parameters, oxidative stress, and inflammation in overweight and obese adolescents. Furthermore, long-term karate training has been linked to significant alterations in immune parameters, including alterations in total and specific leukocyte counts, as well as immunoglobulin concentrations. Furthermore, karate training has been demonstrated to enhance musculoskeletal strength, improve muscle tone, and facilitate postural adjustments, thereby potentially reducing the risk of occupational overuse syndromes. Furthermore, it has been demonstrated to have a beneficial effect on well-being and a reduction in symptoms related to mental health internalisation. Karate training has been linked to increased life satisfaction, which is thought to be mediated by improvements in self-control and emotion regulation. However, it is important to note that karate training does carry a risk of knee injury, particularly anterior cruciate ligament (ACL) rupture, cartilage damage, and meniscus damage. Additionally, long-term karate training has been observed to significantly impact the quantitative ultrasound measurements of the heel bone among advanced practitioners, leading to higher bone density. Among children, karate training has been shown to significantly influence their motor skills.

With the above in mind, the position can be taken that the health effects of karate training are indeed similar to other fighting methods [41,42,43,44,45]. At the same time, it should be noted that not all fighting methods have health benefits. Therefore, modern health discoveries regarding karate are no different from those of other fighting methods, and do not add anything new to the discoveries of the last century [9,10,11,12,13,14,15,16,17,18,19].

### 4.2. Identification of Contradictions

The solution to the problem came from a critical analysis of the source texts, which revealed that existing research findings on karate in the 21st century refer to relatively small research groups. It was found that none of the referenced articles achieved the minimum number of subjects in the given samples, thereby preventing the making of holistic inferences about a given population of karate athletes, including children, adolescents, adults, women, and men. This implies that the inferences drawn from the analysed studies are limited to the individuals under investigation and are, therefore, probabilistic in nature. Consequently, a conclusion was formulated that there is no comprehensive evidence on the health-promoting effects of karate training in relation to the physical and mental health of children, adolescents, and adults. On the other hand, the methodological limitation of this study is that it refers to only one database—Scopus.

However, it should be noted that the situation of inconsistent results due to small samples or a lack of standardised methodology is similar in studies of other fighting arts [46,47,48]. Referring to research into only one fighting method, it is impossible to find contemporary works that meet the requirements of inference on a given population of trainers. As long as researchers do not move away from the generalised inclusion of practitioners of combat sports, martial arts, and self-defence systems, it will not be possible to draw precise conclusions about the population training in a particular fighting method. Accordingly, it was pointed out that the direction of research on the health effects of karate training is a niche direction, which is the cognitive novelty of this article. And the health effects of karate training still remain a generalised issue, rather than the exact specifics of hand-to-hand combat methods that researchers have been studying [9,10,11,12,13,14,15,16,17,18,19].

### 4.3. Limitations and Recommendations for Further Research

It is therefore recommended that interdisciplinary research on large populations of karate athletes be conducted and disseminated. These must be rigorous studies. It is important that continued research be conducted on large and homogeneous groups among children, adolescents, and adults. Training experience in the following groups should be included here: beginner (coloured belts from 9 to 7 kyu), intermediate (coloured belts from 6 to 4 kyu), advanced (coloured belts from 3 to 1 kyu), and master (black belts from 1 dan). Also of great importance will be the experiences of those studied in karate due to their training goals—different for combat sport, martial art, and self-defence system. Therefore, further research should also be focused on training goals. Such research in a systematic way will be able to contribute to the thesis that there is comprehensive evidence of the health-promoting effects of karate training on the physical and mental health of children, adolescents, and adults. Small samples limit the ability to extrapolate the benefits of karate to the general population, pointing to the need for longitudinal studies with greater statistical power. In addition, the need for methodological standardisation is noted. Results to date show a significant gap in the standardisation of methodology. Interdisciplinary efforts to develop more robust research protocols will be important here.

## 5. Conclusions

The health effects of karate training have been documented in the results of anthropological, anthropomotor, biological, medical, and psychological studies. These effects include better immune function, increased motor skills, and greater physical fitness and well-being. However, the studies reviewed are largely limited to small, specific populations, such as elite athletes or children, and therefore cannot be generalised to the broader karate training population. The analysed studies were conducted on small sample sizes and lacked diversity. These limitations make it difficult to draw strong conclusions about the entire karate training population.

Since the current evidence is insufficient to conclusively determine the effects of karate training on health, further research and dissemination of the findings are needed. Future studies should focus on larger, more homogeneous groups of participants, include a variety of skill levels, and examine the physical and psychological health effects of karate training across age groups and training goals (combat sport, martial art, or self-defence system). Research gaps need to be filled, such as the lack of longitudinal studies, the limited examination of gender differences, and the lack of standardised methodologies across studies.

With the above in mind, it was concluded that there is evidence of positive health effects of karate, but long-term health effects are lacking. Longitudinal studies that consider long-term health effects are needed. At the current level of evidence, karate does not stand out more than other forms of physical activity on issues of broader health. In addition, the small amount of contemporary research strictly on karate indicates a research gap and the validity of conducting research in this area.

## Figures and Tables

**Table 1:** Summary of results [27,28,29,30,31,32,33,34,35,36,37,38,39,40].

Date	Journal	Authors	Study Objective	Results	*p*	Doi
2009 Aug 27	*Eur J Appl Physiol*	Doria, C.; Veicsteinas, A.; Limonta, E.; Maggioni, M.A.; Aschieri, P.; Eusebi, F.; Fanò, G.; Pietrangelo, T.	Energetics of karate (kata and kumite techniques) in top-level athletes	The metabolic power was significantly higher in kumite than in kata athletes; aerobic and anaerobic alactic sources, in percentage of the total, were significantly different between gender and disciplines, while the lactic source was similar.	*p* < 0.05	10.1007/s00421-009-1154-y
2012 Oct 1	*Sports Med*	Chaabène, H.; Hachana, Y.; Franchini, E.; Mkaouer, B.; Chamari, K.	Physical and physiological profile of elite karate athletes	There is no significant difference between male and female kata (forms) and kumite (sparring/combat) athletes with regard to aerobic performance; further research is needed concerning differences between kata and kumite athletes and variations based on weight categories.	review	10.1007/BF03262297
2015 Apr 18	*Atten Percept Psychophys*	Muiños, M.; Ballesteros, S.	Sports can protect dynamic visual acuity from aging: A study with young and older judo and karate martial arts athletes	Karate athletes had better DVA than non-athletes; the older adult groups showed a larger oblique effect than the younger groups; the DVA of young karate athletes was superior to that of non-athletes.	*p* < 0.05	10.3758/s13414-015-0901-x
2016 Dec 27 (2017 issue)	*Sport Sci Health*	Mastnak, W.	Karate-based prevention of work-related musculoskeletal syndromes: a study on the possible benefits of martial arts in sports medicine and for occupational health	Karate training strengthens the musculoskeletal system, balances the muscle tone, and helps to readjust the posture.	narrative review	10.1007/s11332-016-0336-3
2018 Jun 26	*Evid Based Complement Alternat Med*	Manzaneque, J.M.; Vera, F.M.; Carranque, G.A.; Rodríguez-Peña, F.M.; Navajas, F.; Blanca, M.J.	Immunological Modulation in Long-Term Karate Practitioners	Karate practitioners exhibited a significantly higher number of total leukocytes, monocytes, and lymphocytes, a higher percentage of monocytes, and greater serum concentrations of IgG and IgM.	*p* < 0.01	10.1155/2018/1654148
2019 Oct 22	*J Phy Edu and Sport*	Altavilla, G.; D’Elia, F.; D’Isanto, T.; Manna, A.	Tests for the evaluation of the improvement of physical fitness and health at the secondary school	Karatekas achieved better results in motor tests, namely, medicine ball throw, shuttle run, and long jump, in comparison to non-athletes.	*p* < 0.05	10.7752/jpes.2019.s5262
2019 Nov 9	*Physiotherapy Quarterly*	Rutkowski, T.; Sobiech, K.A.; Chwałczyńska, A.	The effect of karate training on changes in physical fitness in school-age children with normal and abnormal body weight	Positive changes in physical fitness were demonstrated among children practising karate; significant improvements were observed among children with normal body weight compared to overweight or obese children.	*p* < 0.01	10.5114/pq.2019.86465
2020 Mar 30	*Arch Budo*	Shaw, I.; Schwartzel, D.; Millard, L.; Breukelman, G.J.; Shaw, B.S.	Lower-body strength, power and flexibility in karateka: implications for musculoskeletal health	The elite karatekas were found to have superior strength in their quadriceps and hamstrings, significantly higher quadriceps peak torque values, and a lower time to peak torque for their quadriceps when compared to active karate athletes.	*p* < 0.05	none
2021 Jun 27	*Int J Environ Res Public Health*	Naserpour, H.; Baker, J.S.; Letafatkar, A.; Rossettini, G.; Dutheil, F.	An Investigation of Knee Injury Profiles among Iranian Elite Karatekas: Observations from a Cross-Sectional Study	The most common types of injuries in karatekas were anterior cruciate ligament (ACL) ruptures, articular cartilage injuries, and meniscus injuries.	*p* < 0.0001	10.3390/ijerph18136888
2022 Jan 13	*Front Behav Neurosci*	Potoczny, W.; Herzog-Krzywoszanska, R.; Krzywoszanski, L.	Self-Control and Emotion Regulation Mediate the Impact of Karate Training on Satisfaction With Life	Self-control and reappraisal fully mediate the impact of karate training on subjective well-being; karate training contributes to increasing the well-being of trainees.	*p* < 0.001	10.3389/fnbeh.2021.802564
2022 May 1	*Pediatr Exerc Sci*	de Souza, F.; da Silva, L.A.; Ferreira, G.S.; de Souza, M.M.M.; Bobinski, F.; Palandi, J.; Marcon, C.E.M.; Martins, D.F.; Schuelter-Trevisol, F.; Trevisol, D.J.	Karate Training Improves Metabolic Health in Overweight and Obese Adolescents: A Randomized Clinical Trial	After 12 weeks of karate training in overweight and obese adolescents compared to before the intervention, resting heart rate, high-density lipoprotein cholesterol, and triglyceride levels were reduced; low-density lipoprotein cholesterol levels, protein carbonylation, and nitric oxide levels were reduced; and superoxide dismutase, glutathione, and adiponectin levels were increased after the intervention.	*p* < 0.05	10.1123/pes.2020-0193
2023 Feb 3	*Int J Environ Res Public Health*	Glinkowski, W.M.; Żukowska, A.; Glinkowska, B.	Quantitative Ultrasound Examination (QUS) of the Calcaneus in Long-Term Martial Arts Training on the Example of Long-Time Practitioners of Okinawa Kobudo/Karate Shorin-Ryu	Long-term karate training impacts the parameters of the calcaneus quantitative ultrasound measurements; significantly higher bone density was observed among the black belt holders; long-term practice subjects achieved results far beyond the norm for their age groups.	*p* < 0.05	10.3390/ijerph20032708
2024 Feb 7	*Sports*	Gaweł, E.; Zwierzchowska, A.	The Acute and Long-Term Effects of Olympic Karate Kata Training on Structural and Functional Changes in the Body Posture of Polish National Team Athletes	Age and the number of weekly kata sessions correlate with the ROM of the lumbar spine; long-term musculoskeletal pain (NMQ-6) is the result of compensatory changes that occur in the musculoskeletal structures as a result of elite-level kata training.	*p* < 0.05	10.3390/sports12020055
2024 Feb 22	*Theory and Practice of Phy Cul*	Skorosov, K.K.; Dorontsev, A.V.; Medvedev, I.N.; Razzhivin, O.A.	Influence of karate training lessons on functional parameters of the heart in first-year students	Karate training for six months strengthens the myocardium of the left ventricle, maintaining the optimum of its internal volume, external dimensions, and functionality.	*p* < 0.05	none

*p*—statistical significance.

## Data Availability

The original contributions presented in this study are included in the article. Further inquiries can be directed to the corresponding author.
